# Cooperativity of Stress-Responsive Transcription Factors in Core Hypoxia-Inducible Factor Binding Regions

**DOI:** 10.1371/journal.pone.0045708

**Published:** 2012-09-24

**Authors:** Diego Villar, Amaya Ortiz-Barahona, Laura Gómez-Maldonado, Nuria Pescador, Fátima Sánchez-Cabo, Hubert Hackl, Benjamin A. T. Rodriguez, Zlatko Trajanoski, Ana Dopazo, Tim H. M. Huang, Pearlly S. Yan, Luis del Peso

**Affiliations:** 1 Department of Biochemistry, Universidad Autónoma de Madrid and Instituto de Investigaciones Biomedicas Alberto Sols, Madrid, Spain; 2 Genomics Unit, Centro Nacional de Investigaciones Cardiovasculares (CNIC), Madrid, Spain; 3 Biocenter, Division of Bioinformatics, Innsbruck Medical University, Innsbruck, Austria; 4 Human Cancer Genetics Program, Department of Molecular Virology, Immunology, and Medical Genetics, The Ohio State University, Columbus, Ohio, United States of America; University of Cambridge, United Kingdom

## Abstract

The transcriptional response driven by Hypoxia-inducible factor (HIF) is central to the adaptation to oxygen restriction. Despite recent characterization of genome-wide HIF DNA binding locations and hypoxia-regulated transcripts in different cell types, the molecular bases of HIF target selection remain unresolved. Herein, we combined multi-level experimental data and computational predictions to identify sequence motifs that may contribute to HIF target selectivity. We obtained a core set of *bona fide* HIF binding regions by integrating multiple HIF1 DNA binding and hypoxia expression profiling datasets. This core set exhibits evolutionarily conserved binding regions and is enriched in functional responses to hypoxia. Computational prediction of enriched transcription factor binding sites identified sequence motifs corresponding to several stress-responsive transcription factors, such as activator protein 1 (AP1), cAMP response element-binding (CREB), or CCAAT-enhancer binding protein (CEBP). Experimental validations on HIF-regulated promoters suggest a functional role of the identified motifs in modulating HIF-mediated transcription. Accordingly, transcriptional targets of these factors are over-represented in a sorted list of hypoxia-regulated genes. Altogether, our results implicate cooperativity among stress-responsive transcription factors in fine-tuning the HIF transcriptional response.

## Introduction

Oxygen is essential for the survival of all eukaryotic cells, and metazoans are heavily dependent on this element to meet their large metabolic demands. At the cellular level, 90% of oxygen is consumed in oxidative phosphorylation. Consistent with a central role of oxygen in aerobic metabolism, all metazoan cells respond to an imbalance between demand and supply of oxygen (hypoxia) by activating a gene expression program aimed at restoring oxygen supply and reducing its consumption. The cellular response to hypoxia is mainly controlled by the evolutionarily conserved Hypoxia Inducible Factor (HIF) family of basic helix-loop-helix transcription factors. HIFs are heterodimers of a beta subunit (HIFβ, also known as ARNT), and an alpha subunit (HIFα) [1]. While ARNT levels are not sensitive to oxygen, both HIFα stability [2] and its transcriptional activity [3] are regulated by oxygen-dependent hydroxylation [4]–[6]. Under oxygen restriction, HIFα subunits escape proteasomal degradation, heterodimerize with HIFβ subunits and translocate to the cell nucleus, where they bind the RCGTG consensus sequence (termed Hypoxia Response Element, HRE) within regulatory regions of target genes, leading to their transcriptional activation in hypoxia [7]. Mammals present three isoforms of HIFα (HIF1α, HIF2α and HIF3α) that differ in their tissue distribution, HIF1α being the more ubiquitous and best characterized [8].

A large number of studies focusing on single genes have identified individual HIF targets that, collectively, account for the functional responses to hypoxia, mainly metabolic adaptation and induction of angiogenesis [7]. More recently, works employing HIF1α and HIF2α chromatin immunoprecipitation coupled to genomic microarrays (ChIP-chip) or high-throughput sequencing (ChIP-Seq) have addressed the genome-wide identification of HIF binding locations [9]–[12], thereby improving the existing knowledge on the HIF-modulated transcriptome and largely confirming the RCGTG HIF binding consensus. Additionally, these studies have provided important insights into the global properties of HIF1 binding and transactivation. First, these works reported a significant association between the presence of a HIF binding site (HBS) and hypoxic induction of the neighboring genes. The same trend was not found for genes repressed by hypoxia, suggesting that hypoxia-mediated repression is largely indirect or HIF-independent [9], [12], [13]. Furthermore, they have clearly shown that only a small subset of about a hundred of all RCGTG-containing genes is robustly regulated by hypoxia. Hence, and in agreement with work on other transcription factors [14], HIFs bind a small proportion of potential binding sites, albeit the basis of their binding and target selectivity are incompletely understood.

Understanding the mechanisms that explain HIFs transactivation selectivity is of paramount importance to expand our knowledge on transcriptional regulation and to improve the sensitivity and specificity of genome-wide efforts to characterize the HIF transcriptional response. DNA accessibility of transcription factor binding sites (TFBSs) can clearly contribute to binding selectivity [15]. For HIFs, recent evidence includes enhanced HIF1 and HIF2 binding to normoxic DNAse hypersensitivity sites [12] and enrichment of HIF1 binding in the proximity of genes with a “permissive” transcriptional state in normoxia, as evidenced by significant basal expression [11]. Additionally, DNA methylation has been also shown to modulate HIF1 binding, as originally demonstrated for the 3′ enhancer of the erythropoietin gene [16], [17]. A further mechanism that can impact target selectivity is direct or indirect cooperativity between transcription factors (TFs). Models of direct cooperativity have been mainly derived from developmental enhancers, and include the strict enhanceosome model [18], where cooperative occupancy occurs through extensive protein-protein interactions between TFs or common cofactors, and the more flexible billboard model [19], which suggests that enhancers contain submodules that interact independently or redundantly with promoters. Conversely, indirect cooperativity is based on the equilibrium competition between nucleosomes and DNA-binding proteins, thereby not requiring protein-protein interactions [20]. In the case of HIF-mediated transcription, the binding of cooperating transcription factors has been demonstrated for several target genes. In particular, HIF-mediated expression of the erythropoietin gene requires an adjacent HNF4 binding site [21], both GATA2 and AP1 binding sites are necessary for epithelial induction of *ET-1* under hypoxia [22], and *PAI-1* induction by hypoxia has been linked to cooperative promoter activation by CEBPα, HIF1α and EGR-1 [23]. Other examples include cooperation with Smads [24], Sp1 [25] or CREB [26]. Additionally, USFs have been shown to complement HIF binding either at neighbouring (*LDHA* promoter) or identical sites (*BNIP3*) [27], while collaboration with ETS transcription factors has been proposed to play a role in HIF2α target selectivity [28], [29]. Recent genome-wide approaches relying on experimental [9]–[11] and computational [13], [30] identification of HIF binding sites have reported overrepresented transcription factor binding sites in the flanking sequences that might be indicative of transcriptional cooperativity. However, significant differences exist in the overrepresented TFBSs predicted in each study, and the functional significance of these enriched motifs remains unclear.

Gene expression profiling indicates that the expression of thousands of genes changes with hypoxia, with vast cell-type differences in the specific genes being regulated [31]–[38]. HIF1α ChIP-chip binding locations have been reported in cell lines of diverse tissue origin, namely HepG2 hepatocarcinoma cells [9], MCF-7 breast cancer cells [10] and U87 glioma cells [11], showing differences in the binding sites identified in each experiment. In previous studies we integrated microarray expression profiling experiments and HIF binding site predictions in a core set of tissue-independent HIF target genes [13]. To further investigate the selectivity of HIF1 binding, in this work we conducted HIF1α ChIP-chip in cervical carcinoma HeLa cells and observed largely non-overlapping binding locations with previous studies. To explore the role of cooperativity in HIF target selection, we integrated HIF1 alpha ChIP-chip binding locations across cell-types with a meta-analysis of gene expression profiles of cells exposed to hypoxia [13]. Computational prediction of enriched transcription factor binding sites in this integrated set suggested several stress-responsive transcription factors as potential HIF1 collaborators. Experimental validation of these predictions in cell-based reporter assays indicates that binding sites for stress-responsive transcription factors other than HIFs, such as CEBPs, contribute to cooperative hypoxic activation of individual targets.

## Materials and Methods

### Gene-expression Profiling Analysis

Gene profiling experiments of hypoxic cell cultures were downloaded from the Gene Expression Omnibus database (GEO, http://www.ncbi.nlm.nih.gov/geo/) [39]. The average raw signal from biological replicates was used in the analysis. When fifty percent of the replicates had null values the average signal was set to null. All probes mapping to a particular locus were considered independently. A gene (identified by a particular probe) was recorded as having no basal expression when the signal for the probe under normoxic conditions had a null value. A gene (probe) was considered to be induced by hypoxia when the log-ratio of the hypoxic over normoxic signal values exceeded by 2.6 standard deviations the average log-ratio of all the probes in the array. Genes (probes) with a null normoxic value and not-null hypoxic values were also considered as induced by hypoxia. The presence and absence of conserved RCGTG motifs at each locus was determined as described previously [13].

### Cell Culture

HeLa cells were maintained in Dulbecco’s modified Eagle medium supplemented with 100 units/ml penicillin, 100 µg/ml streptomycin and 5% (v/v) fetal bovine serum. Cells were grown at 37°C and 5% CO_2_. Hypoxic treatments were carried out in sealed chambers flushed with a 1%O_2_/5%CO_2_/94%N_2_ gas mixture (Billups-Rothenberg, Inc.; CA, USA).

### Chromatin Immunoprecipitation

Chromatin immunoprecipitation was performed as previously described [40]. Briefly, 10^7^ HeLa cells were subjected to hypoxia (1% oxygen) for six hours or left untreated (normoxic conditions, 21% oxygen). Following treatments, cells were crosslinked with 1% formaldehyde for 12 min at 4°C. Cross-linking was stopped by the addition of glycine (0.125 M final concentration). Cell lysis was achieved by scraping in 1 ml of lysis buffer (1% SDS, 10 mM EDTA, 50 mM Tris/HCl, pH 8.1, and a protease inhibitor cocktail, Roche). Cell lysates were incubated on ice for 10 min and then sonicated to shear DNA to fragments between 200 and 1500 bp. Only experiments that showed homogeneous sonication across all samples (from normoxia and hypoxia treatments) were continued. 50 *µ*l of each sample was stored (input), while 100 *µ*l were diluted in 1 ml of immunoprecipitation buffer (1% Triton X-100, 2 mM EDTA, 150 mM NaCl and 20 mM Tris/HCl, pH 8.1). Lysates were precleared with 200 *µ*g of a Salmon Sperm DNA/Protein A agarose 50% slurry (Upstate Biotechnology, Lake Placid, NY, U.S.A.) for 1 h at 4°C; and then immunoprecipitated twice, initially with whole rabbit serum for 6 h (IgG control) and then overnight at 4°C with a polyclonal anti-HIF1 alpha antiserum (Abcam, ab2185). Immunocomplexes were recovered by addition of 400 *µ*g of Salmon Sperm DNA/Protein A agarose 50% slurry, and then sequentially washed in Low Salt Wash Buffer (0.1%SDS, 1%Triton X-100, 2 mM EDTA, 20 mM Tris/HCl, pH 8.1, and 150 mM NaCl), High Salt Wash Buffer (0.1% SDS, 1% Triton X-100,2 mM EDTA, 20 mM Tris/HCl, pH 8.1, and 500 mM NaCl), LiCl buffer (0.25 M LiCl,1% Nonidet P40, 1% deoxycholate, 1 mM EDTA and, 10 mM Tris/HCl, pH 8.1), and twice in TE buffer (10 mM Tris, pH 8.0, and 1 mM EDTA). Elution of protein-bound DNA was performed twice with 1% SDS 0.1 M NaHCO3. Eluates were pooled, and crosslinking was reversed by the addition of 200 mM NaCl (final concentration) and overnight incubation at 65°C. Protein and RNA were removed by the addition of proteinase K (30 µg/sample) and RNAse (200 mg/µl) for 2 hours at 42°C, and immunoprecipitated DNA was purified by phenol-chloroform extraction and ethanol precipitation. Successful ChIP was assayed by standard PCR using two primer pairs, targeting the functional EGLN3 HRE and a control region in the same *locus* [40].

### Chromatin Immunoprecipitation on Microarray

The ChIP-chip method was carried out as previously described [41]. First, purified DNA from chromatin immunoprecipitation was amplified by ligation-mediated PCR. DNA ends were extended by incubation with T4 DNA polymerase (New England Biolabs), and blunted DNA was ligated to pre-annealed oligonucleotide linkers (JW102 gcggtgacccgggagatctgaattc and JW103 gaattcagatc) using T4 DNA ligase (New England Biolabs), and subsequently amplified by two rounds of PCR using JW102 as primer, to yield 2–5 µg of amplified DNA. An aliquot of this material was run in a microfluidics platform (Agilent 2100 Electrophoresis Bioanalyzer) to accurately measure size distribution of amplified material and discard gross amplification bias. Additionally, quantitative PCR against both a Hypoxia-Response Element (HRE) in the EGLN3 locus [40] and a control negative region in the same locus was routinely performed to assess loss of enrichment during amplification.

Amplified DNA from normoxic and hypoxic chromatin immunoprecipitation samples was labelled with Cy3 and Cy5 fluorescent dyes, respectively, and hybridized to microarrays following guidelines from the microarray manufacturer (Agilent Mammalian ChIP-on-chip Protocol v.10). Hybridized microarray slides were scanned in an Agilent DNA microarray scanner (Agilent Technologies, CA, USA) at 5 µm resolution, and acquired microarray images were quantified with GenePix 6.0 software (Molecular Devices, CA, USA). A total of six hybridizations were conducted, corresponding to four biological replicates. The two technical replicates were dye-swap experiments, where normoxic samples were labelled with Cy5 and hypoxic samples with Cy3.

### Analysis of ChIP-chip Data

A custom alternative promoter microarray was used for ChIP-chip hybridizations [42]. Probes in the array cover 34000 known or putative promoters representing about 7000 human genes, and tile a region from −200 to +200 of known or predicted transcription start sites, with an average probe spacing of 80 bp. Genomic coordinates of the probes in the array (hg17, May 2004) were updated to the hg19 assembly using the alignment tool Exonerate [43] with 97% sequence identity. Probes having non-unique matches to this version of the Human Genome were excluded from ChIP-chip analysis.

The R/Bioconductor packages “Ringo” and “limma” were used to analyze ChIP-chip readouts [44], [45]. Limma analysis was performed after normalization of ChIP-chip data with the variance-stabilizing method. A separate linear model was fitted to each biological replicate, which comprised a single readout or two in the case of dye-swap experiments, and these models were averaged to obtain a single linear model that includes estimation of moderated t-statistic p values. The Benjamini-Hochberg correction for multiple comparisons (False Discovery Rate) was applied to these p values. Ringo analysis was performed essentially as described [46], using the parameters indicated below. Raw data were again normalized with the variance-stabilizing method. First, for the calculation of the average smoothed signal across replicates, we used: winHalfSize = 100 (based on probe density and spacing in the array) and quant = 0.75. To obtain a threshold intensity value for bound probes, a 0.99 quantile was used as upper bound for the null distribution. For the identification of ChIP-enriched regions on the smoothed signal, distCutOff = 200 (maximum probe spacing within a single ChIP-enriched region) and minProbesInRow = 4 (minimum number of probes per region) were used. Minor modifications to Ringo functions “cherByThreshold” and “findChersOnSmoothed” were made to take into account probe-wise p values (as previously calculated by limma) for ranking of ChIP-enriched regions. Specifically, ChIP-enriched regions found by Ringo were required to harbour one or more probes with an FDR-adjusted p value lower than 0.02 (2% false discovery rate). Finally, ChIP-enriched regions in poorly covered regions of the array (having less than 8 total probes, 4 inside the region and 4 surrounding it) and those mapping to repetitive regions were identified with in-house Perl scripts and taken out of the analysis.

The microarray experiments described above have been deposited in ArrayExpress under accession number E-MEXP-3499.

### Quantitative PCR

Purified DNA from chromatin immunoprecipitation samples was used in quantitative PCR with SYBR green-based detection (Applied Biosystems) following the manufacturer’s recommendations. Primers targeting candidate ChIP-enriched regions were designed with Primer Express software v2.0 (Applied Biosystems) and Primer3 (http://frodo.wi.mit.edu/primer3/). All measurements were carried out in triplicate. Threshold cycle (Ct) values for each sample were interpolated in a standard curve of input DNA dilutions to obtain % of input absolute values. Enrichment of HIF1 alpha binding to target loci was calculated as the ratio of the amounts of target sequence (measured as % of input) detected in hypoxic vs noxmoxic ChIP samples (% of input hypoxia/% of input normoxia). For validation of ChIP-chip candidates, three negative control regions (in the EGLN3, IRS4 and HIPEV1 loci, [13]) were used to estimate an average background enrichment, and a 90% confidence interval was applied on these values to set a threshold for successful validation of candidate regions.

### Obtaining a High-confidence Set of Core HIF Binding Regions and a Background Set of Control Regions

Custom scripts written in Perl were used to indentify evolutionarily conserved HIF binding sites (HBSs) within ChIP-chip regions and to select HBSs that showed evolutionary conservation. Conservation of the HIF binding consensus RCGTG in four mammalian species, including mouse, was required for the evolutionary conservation filter. HBSs were further selected to map to genes robustly induced by hypoxia, as indicated by the results of a meta-analysis of gene expression profiling experiments in hypoxic cell cultures [13], using a p value threshold of 0.02 (FDR). Finally, HBS coordinates were extended into HIF binding regions (HBRs) that spanned surrounding non-coding conserved sequences, as determined by >50% presence of phastCons elements [47], and the corresponding multiple sequence alignment of each HBR was retrieved. Multiple sequence alignments were downloaded from the UCSC Genome Browser’s Table Browser [48].

To obtain a set of background (control) genomic regions, custom perl scripts were used to exploit the microarray meta-analysis results for the identification of genes harbouring conserved RCGTG motifs but that are unlikely to be modulated by hypoxia. To this end, gene *loci* that contained conserved RCGTG motifs in their non-coding sequences were first selected. For these genes, each of their probes was examined, and only genes for which all of their associated probes exhibited a mean fold value within 0.25 standard deviations of the global mean in each of the 19 datasets employed in the meta-analysis were considered as not induced by hypoxia. The selected coordinates of conserved RCGTG motifs mapping to these *loci* were extended as previously described for the set of core HIF binding sites. Genomic regions from this collection were further selected to match the frequency of genomic locations (relative to the TSS) found in the core HBR set. Briefly, Perl scripts were used to annotate core HBRs as promoter, 5′UTR, intronic or 3′UTR genomic locations and to choose, from the whole collection, a random sample according to the proportions of genomic locations found in the core HBR set. Similarly as with the set of core HIF binding regions, multiple sequence alignments corresponding to the selected control regions were retrieved.

### In silico TFBS Prediction

*In silico* transcription factor binding site predictions were carried out employing custom scripts written in Perl. Position-weight matrices (PWMs) collections were downloaded from the public databases JASPAR (2010 release) and Transfac (7.0 version) [49], [50]. Raw frequency matrices were transformed into log-odd matrices to take into account the background nucleotide frequencies found in the whole collection of HIF binding regions (core and background together), and the information content of each matrix position was calculated as proposed by Stormo [51]. The formulae used for these calculations are detailed below.

PWM conversion:

The weight of base b in position i is calculated as:
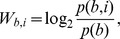
where p(b,i) is the corrected probability of base b in position i and p(b) the background probability of base b.

The corrected probability is obtained from the raw matrix by adding a pseudocount:
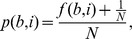
where f(b,i) are the counts of base b in position i and N the number of sites used to construct the matrix.

Information content calculation:
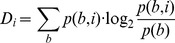



Subsequently, log-odd matrices were used to screen core HIF binding regions and genomic-matched background regions for the presence of putative TFBSs or other sequence motifs. Perl scripts were employed to split each sequence into overlapping fragments of length equal to that of the PWM under analysis. For each fragment, a score value was calculated by summing up the log-odd frequencies obtained by substitution of nucleotides found in the fragment in the corresponding position of the PWM. The contribution of each base to the score was weighted by the information content of its position in the matrix, as detailed below.

Score calculation:
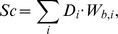
where D_i_ is the information content of position i and W_b,i_ the log-odd weight of base b in position i.

Finally, the resulting score was normalized by subtraction of the minimum score and division by the score range, and compared with a threshold value. Fragments showing a score above the threshold were considered as putative TFBSs, and the evolutionary conservation of nucleotides in each motif was evaluated for matrix positions with information content over 60%. Putative TFBSs showing evolutionary conservation in four mammalian species (including mouse) and whose score was over the threshold value were recorded as present (respresented as 1). Otherwise, they were considered absent from the analyzed sequence (represented as 0).

For each PWM, the three strategies proposed in MATCH [52] were used for the calculation of threshold values. The minFN strategy aims at minimizing false negative predictions (low stringency), and was obtained by setting the threshold value that detects 90% of cases in a randomly generated sample of sequences in which the probability of nucleotides at each position is dictated by the matrix frequencies. Conversely, the minFP threshold focuses on minimizing false positives (high stringency), and its calculation is based on the assumption that coding sequences in the Genome are impoverished in functional TFBSs. We used the threshold value that results in a single hit (on average) per 10000 bp when the matrix is used on all human coding sequences. Finally, the minSum threshold was obtained by estimating the false positive and false negative rate for all threshold values between minFN and minFP, and then choosing the value that minimizes the sum of both (medium stringency).

### Statistical Analysis of Enriched TFBSs

Fisher’s exact test was used to identify PWMs showing significant enrichment in the set of core HIF binding regions *versus* the background collection. In particular, we considered significant PWMs with a p value lower than 0.05. No correction for multiple comparisons was applied to these p values.

Additionally, the Weka machine learning workbench [53] (3.6 version) was used to identify the most informative PWMs, that were better able to distinguish core HIF binding regions from the background set. To this end, the correlation-based feature selection variable selection procedure was used. The algorithm was run 10 times using stratified 10-fold cross-validation in each iteration. Finally, the number of times that each variable had been selected was recorded. This number ranges from zero (never chosen) to a hundred (corresponding to every cross-validation fold and every iteration).

### Plasmid Construction

Human genomic DNA extracted from HeLa cells was used as template for PCR amplification of the *CA9* promoter region (hg19 coordinates chr9∶35673508–35673956), which was subcloned into the pGL3-Basic plasmid (Invitrogen). The Human *GYS1* reporter construct (hg19 chr19∶49496421–49496978) has been previously described [54]. The mouse *LDHA* promoter construct (mm9 chr7∶54101027–54101258) and the derived HRE and CREB binding site mutations were a kind gift from Peter Ratcliffe [26]. The remaining HRE, predicted AP1 or CEBPB binding sites and control mutations were generated by site-directed mutagenesis, employing QuikChange Site-directed mutagenesis kit (Stratagene). The introduced mutations are detailed in Table S1. The identity of all constructs was verified by sequencing.

### Reporter Assays

Reporter assays were performed using the human cervical-carcinoma cell line HeLa. Cells were seeded on six-well plates (2.5·105 cells/well) 6 h prior to transfection. Per well, a 4.5 µg DNA mixture containing 1.5 µg of the indicated reporter construct or empty plasmid and 0.25 µg of a plasmid encoding for Renilla (sea pansy) luciferase under the control of a null promoter (Promega, Madison, WI, U.S.A.) was used for transfection using the calcium phosphate method. 16 h after transfection, cells where washed, replated in 24-well plates, and incubated in normoxia, in the presence of DMOG (dimethyloxaloylglycine, 500 µM) or in hypoxia for an additional 16 hours. After treatments, cells were lysed and the firefly and Renilla luciferase activities of the lysate were determined using a dual-luciferase system (Promega, Madison, WI, U.S.A.). The firefly luciferase activity was normalized to that of Renilla luciferase. Each experimental condition was assayed in duplicate. Hypoxia or DMOG fold induction values for each experiment were analyzed by repeated measures ANOVA with a Dunnet posthoc correction, comparing values of the wild-type promoter construct to each of the introduced mutations.

### Gene-set Enrichment Analysis

Gene-set enrichment analysis (GSEA) was carried out as previously described ([55] and http://www.broadinstitute.org/gsea/index.jsp). We employed a ranked list of core hypoxia-regulated genes derived from a meta-analysis of 16 hypoxia gene expression experiments [13], where genes are sorted by their mean hypoxic induction across cell lines/tissues represented in the meta-analysis. We studied the distribution of transcription factor targets in this list employing 3000 gene-sets from the GSEA molecular signatures database, which includes experimentally derived lists of targets for specific transcription factors. GSEA analysis provides and enrichment score (ES) for each gene-set across the ranked list of hipoxia-responsive genes. In order to compare several gene-sets, enrichment scores are normalized to produce NES values (normalized enrichment scores). Comparison with NES values obtained from random gene-sets allows estimation of statistical significance. We used an FDR-adjusted p-value of 0.05.

## Results

### 1. Basal Gene Expression is Necessary but not Sufficient for HIF Target Selection

**Figure 1:**
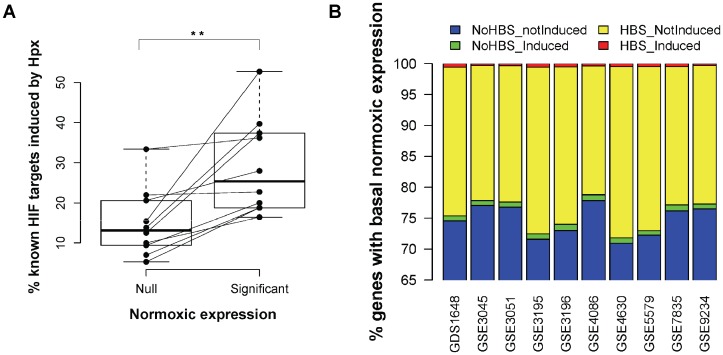
Basal gene expression is not sufficient for HIF transactivation. (**A**) A list of well-characterized HIF target genes (from ref. 7) present in individual gene expression profiling (microarray) datasets (see B for GEO IDs) were categorized according to their basal (normoxic) expression level into two groups: Null, no detectable basal expression; Significant, detectable basal expression. In addition, for each microarray experiment, HIF-target genes were further classified into those whose expression was significantly induced by hypoxia (ratio hypoxia/normoxia greater than 2.6Sd above the mean) and non-responsive genes. The graph represents the percentage of HIF target genes in each category that were induced by hypoxia. Each pair of joined dots represents the data from a single microarray experiment. Box and whisker plots represent the distribution of values in each category. **: p<0.01 (Wilkoxon matched test) (**B**) For each of the indicated microarray datasets (GEO identifiers in horizontal axis), we identified all the genes showing a significant basal (normoxic) expression. Then, we classified them according to their response to hypoxia (“Induced” and “notInduced”, see A) and the presence of conserved RCGTG motifs in their regulatory regions (“HBS” and “NoHBS”, respectively). The graph depicts cumulative percentages (vertical axis) of genes in each of the four combinations of the two categories: no conserved HIF binding motifs and no hypoxic induction (blue, NoHBS_notInduced), no conserved HIF binding motifs but hypoxic induction (green, NoHBS_Induced), conserved HIF binding motifs but no hypoxic induction (yellow, HBS_notInduced) and conserved HIF binding sites and hypoxic induction (red, HBS_Induced).

Previous studies have proposed that chromatin accessibility and basal gene expression mediate HIF target selectivity [11], [12]. In order to independently assess the contribution of this mechanism to HIF target selection, we exploited publicly available genome profiling experiments of hypoxic cell cultures [13] to look at the association of basal expression and hypoxic induction. We analyzed the basal expression of a list of well-characterized HIF targets [7] and correlated it with their response to hypoxia. In agreement with previous reports [11], [12], we found that the response to hypoxia, scored as the percentage of HIF-target genes induced by the treatment, was significantly higher among genes that were already expressed in the basal (normoxic) condition (Figure 1A, p<0.01 Wilcoxon matched test). Moreover, when the HIF target genes across all datasets were pooled and categorized according to their basal expression and response to hypoxia, the distribution was significantly different to that expected by chance (Table S2, p<0.001 Chi-square test). These results further suggest that chromatin accessibility contributes to HIF target selectivity by modulating the availability of RCGTG motifs. However, given the large number of genes with basal (normoxic) expression and the widespread distribution of the RCGTG motifs, it is expected that many RCGTG motifs would lie within open chromatin regions. To look at the contribution of chromatin accessibility in more detail, we next studied the response to hypoxia of all the genes with detectable normoxic values represented in each array. To this end, within the group of genes with basal gene expression, we identified those harbouring conserved HIF-binding motifs in their non-coding sequences (Figure 1B, HBS) and categorized them according to their induction by hypoxia. For each microarray dataset, the large majority of genes harbouring a conserved RCGTG motif were not induced by hypoxia (Figure 1B, yellow bar segments) in spite of proximity of the motif to genes with significant normoxic expression. This observation strongly suggests that although basal gene expression correlates with hypoxia inducibility of a gene, additional mechanisms are needed to specify HIF target selection.

### 2. Comparative Analysis of HIF1 Alpha Binding Locations in Cell Lines of Diverse Tissue Origin

The binding of additional transcription factors in proximity of HIFs constitutes a plausible mechanism that could contribute to HIF target selection. In this regard, previous works have addressed the identification of sequence motifs overrepresented in collections of HIF binding regions obtained from ChIP-chip datasets or combinations of computational predictions and gene expression profiling experiments [9], [10], [30]. A recent work failed to identify clearly overrepresented sequences [9], while the predictions reported in two other studies showed very small overlap [10], [30]. On the other hand, the wealth of HIF1 alpha binding and hypoxic gene expression data obtained in different cell types provides a unique opportunity to construct integrated sets of HIF1 binding sites that may overcome the limitations of datasets based on a single experiment. In order to study the role of cooperativity in HIF target selectivity, we determined the genome-wide pattern of HIF1 alpha binding sites in cervical carcinoma HeLa cells and compared our results to previously published HIF1 ChIP-chip experiments in hepatocellular carcinoma HepG2 cells [9], breast cancer MCF-7 cells [10] and U87 glioma cells [11], as detailed below.

**Figure 2:**
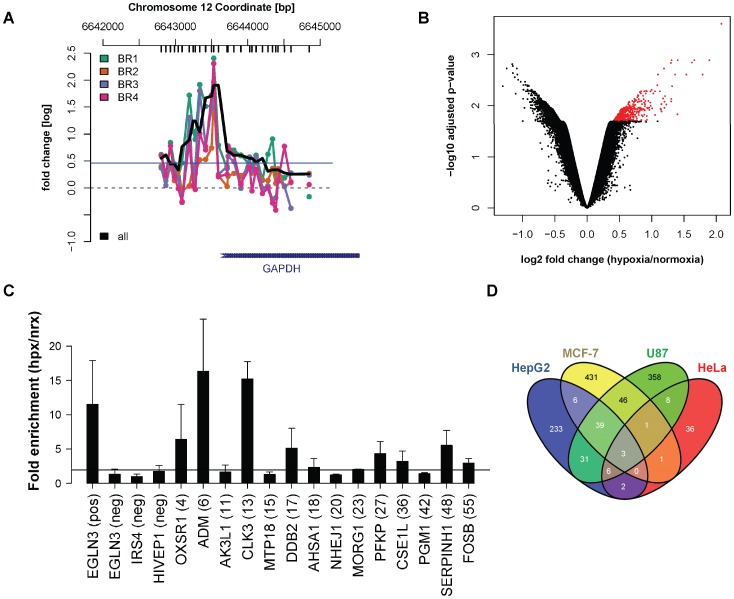
Comparative analysis of HIF1A ChIP-chip data in cell lines of different tissue origin. (**A**) Visualization of HIF1A ChIP-chip readouts for the GAPDH promoter region in HeLa cells. The plot represents normalized intensities (log fold change hypoxia/normoxia, vertical axis) along the hg19 genomic coordinate (horizontal axis). Vertical black bars (top of the graph) mark the center position of array probes. The signal of four independent biological replicates is indicated in different colors (BR1 to 4). The smoothed black line corresponds to the averaged signal across replicates. The horizontal blue line indicates the intensity threshold for bound probes. (**B**) Volcano plot of HeLa ChIP-chip data (linear model across the four biological replicates). Spots in the plot correspond to individual probes in the array. Probes significantly enriched in hypoxic samples (p<0.02, FDR) are highlighted as red spots. (**C**) Quantitative PCR validation of HeLa ChIP-chip candidates. The official Gene Symbol corresponding to each region is indicated in the horizontal axis. Four control regions (one positive and three negative) were used as reference to estimate the successful validation of the indicated ChIP-chip candidates (candidate ranks in parenthesis). Bars represent the average fold enrichment in hypoxic versus normoxic ChIP samples (vertical axis), as obtained in three independent experiments. Error bars represent the standard deviation. The horizontal black line indicates the threshold for successful validation (90% confidence interval). (**D**) 4-way Venn diagram indicating the overlap of HIF1A bound regions as reported by ChIP-chip studies in hepatoma (HepG2 cells, ref. 9), mammary gland (MCF-7 cells, ref. 10), glioma (U87 cells, ref. 11) and cervix (HeLa cells, this study) origin.

For the determination of HIF binding sites in HeLa cells, we performed HIF1α chromatin immunoprecipitation in HeLa cells exposed to normoxia or hypoxia (1% oxygen) for six hours. Amplified samples from normoxic and hypoxic cells were competitively hybridized to a proximal promoter microarray that tiles a subset of human 7000 genes [42]. ChIP-chip data was analyzed with the R/Bioconductor packages Ringo [44] and limma [45] to identify hypoxic HIF1-bound genomic regions. Stringent statistical thresholds (2% FDR) were applied to normalized signals from four biological replicates (Figure 2A, all). ChIP-enriched regions were required to harbour four or more probes above background average signal (Figure 2A, blue horizontal line) and one or more probes robustly induced by hypoxia in a linear model of the four replicates (Figure 2B, red dots, 2% FDR). This analysis produced a ranked list of 57 HIF1 binding regions (Table S3), spanning the coordinates of previously characterized HIF binding sites [7] and including many potentially novel HIF1 binding sites and HIF1 targets. Quantitative PCR validation of the ChIP-chip results in independent HIF1 alpha chromatin immunoprecipiations confirmed hypoxic enrichment of the majority of tested candidates (Figure 2C).

Next, we compared HIF1α ChIP-chip predictions in the four cell lines by analyzing the overlap of reported binding locations (Figure 2D). The majority (36 sequences) of HeLa HIF1α binding locations did not overlap with ChIP-chip results obtained in other cell types, although many were also found in at least one of the previous reports. A similar trend was observed taking any of the other studies as reference, suggesting that most HIF1 binding is cell-type specific. To test the role of cooperativity in dictating HIF1 target selection, we focused on HIF1α ChIP-chip binding locations common to two or more studies as a bona-fide set of core HIF1 binding regions. Analysis of evolutionary conservation in these sequences, defined as RCGTG motifs within PhastCons elements [47] and conserved in at least four mammalian species including human and mouse, showed a strong enrichment of conserved sequences in the core set of common HIF1 binding sites, versus those found uniquely in a single ChIP-chip study (Table S4). Since HBSs identified in more than one study are more likely to correspond to functional sites, this analysis suggest that evolutionary conservation of HIF binding motifs can be predictive of functionality as has been shown for other TFBSs [56], [57].

### 3. Binding Sites for Diverse Stress-responsive Transcription Factors are Enriched in *bona fide* HIF Binding Regions

**Figure 3:**
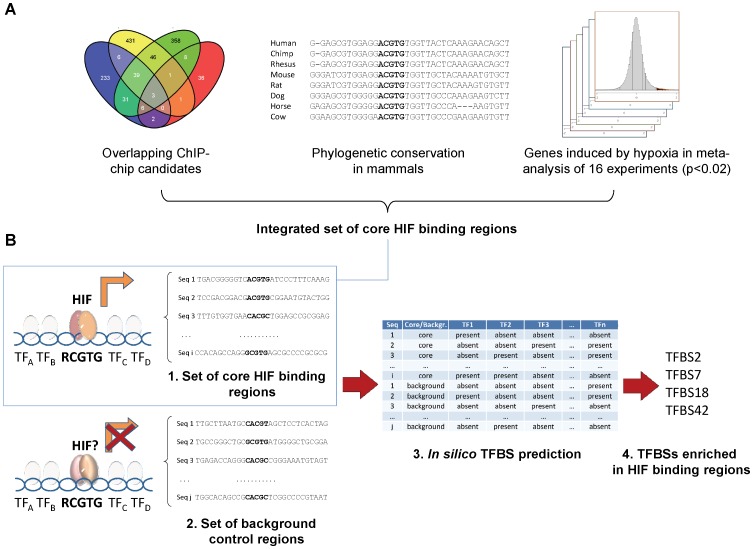
Integrative strategy for prediction of cooperativity in HIF binding regions. (**A**) HIF1 binding locations common to at least two out of four different ChIP-chip studies in HeLa, HepG2, MCF-7 and U87 cells (left), mammalian sequence conservation of the HIF binding regions (center) and regions close to genes robustly induced in hypoxia in a meta-analysis of 16 gene expression experiments (right) were integrated into a set of *bona-fide* core HIF binding regions (**B**) Stepwise diagram for prediction and validation of TFBSs enriched in core HIF binding regions: collection of a set of core HIF binding regions and a background set of control sequences (left), *in silico* prediction of transcription factor binding sites present or absent in the sequences of core and background sets (center), statistical analyses of enriched TFBSs in sequences from the core set (right, top) and experimental validation of these predictions (right, bottom).

We employed the previous set of core, *bona fide* HBSs to computationally identify enriched TFBSs that could be indicative of transcription factor cooperation. To focus on binding locations for which there is evidence of transcriptional modulation of nearby genes in hypoxia, we sought to combine the core set of HIF1 binding locations with HIF transactivation data. To this end, we employed our previous microarray meta-analysis study [13] of 16 gene expression profiling experiments comparing normoxic and hypoxic cell cultures. This integrated gene-expression dataset was used to select, from the binding dataset, HIF1 binding locations that mapped close to genes showing robust hypoxic induction (p<0.02, false discovery rate) (Figure 3A, right). Lastly, and in order to reduce the number of spurious predictions in *in silico* sequence analyses, we focused on HIF binding sites whose sequence showed evolutionary conservation in mammalian species (Figure 3A). These selection criteria produced an integrated set of core HIF binding sites (Table S5). A gene annotation enrichment analysis of the sites in this integrated set revealed enriched annotation terms clearly associated with functional responses to hypoxia, such as glycolysis, 2-oxoglutarate dioxygenase activity and glycogen metabolism [7], [58] (Table S6), strongly suggesting that it faithfully represents *bona fide* HIF binding locations.

We next sought to identify putative TFBSs enriched in the vicinity of the selected core HIF binding sites (Figure 3B). For this purpose, we obtained the sequences flanking each HBS (Table S5). The length of flanking non-coding sequences was based on evolutionary conservation, as indicated by genomic annotation of PhastCons elements [47]. Statistical assessment of sequence motif enrichment in this set of sequences requires comparison with a background set, the election of this set greatly influencing the results of the analysis [59]. We constructed a set of sequences resembling those in the set of core HIF binding regions by screening the non-coding Human Genome for evolutionarily conserved HIF binding consensus sequences, and extended these motifs to span the flanking conserved sequence (Figure 3B and Figure S1). From this set, we selected regions that are unlikely to be transcriptionally modulated by hypoxia, as judged by no differential expression in any of the 16 hypoxia experiments included in our previously reported genome profiling meta-analysis [13]. Finally, a subset from these sequences was chosen that matched the genomic locations and base composition found in the core set (Figure S1). We thereby obtained a custom set of circa 3500 background sequences containing a RCGTG HIF binding consensus.

The sequences in the core HIF binding regions and background sets were screened for TFBSs employing the mammalian position-weight matrixes from the public Transfac 7.0 and Jaspar (2010 release) databases and custom scripts based on the MATCH algorithm [52], recording the presence or absence of a total of 605 sequences motifs in each sequence by using three different stringencies (Table 1). In order to reduce spurious hits, we only considered as positive hits those motifs that were conserved in mammalian species. Fisher’s exact test was applied to these datasets to identify motif predictions enriched in the set of core HIF binding regions. Enriched motifs were consistently found across different stringencies and database sets. In addition to HIF PWMs, we found a significant enrichment for PWMs associated to CREB1, FOS/AP1 and NFY (Table 1).

**Table 1:** Enriched TFBSs in core HIF binding regions (Fisher’s exact test).

PWM Collection	Stringency	Overrepresented PWM	Hits	Transcription factor	P value
JASPAR CORE 2010	minFN (low)	MA0033.1_FOXL1	53	FOXL1	0,001
		MA0259.1_HIF1A::ARNT	54	**HIF1**	0,0076
	minFP (high)	MA0018.2_CREB1	7	**CREB1**	0,0203
		MA0060.1_NFYA	9	**NFYA**	0,0234
		MA0259.1_HIF1A::ARNT	44	**HIF1**	6E−15
	minSum (intermediate)	MA0032.1_FOXC1	36	FOXC1	0,0065
		MA0060.1_NFYA	14	**NFYA**	0,0218
		MA0099.1_Fos	20	**FOS**	0,0305
		MA0259.1_HIF1A::ARNT	52	**HIF1**	3E−06
JASPAR PHYLOFACTS 2010	minFN (low)	PF0014_TGACGTCA	4	**FOS/AP1**	0,0377
		PF0032_TGASTMAGC	3	NF-E2	0,0268
	minFP (high)	PF0014_TGACGTCA	4	**FOS/AP1**	0,0445
		PF0032_TGASTMAGC	3	NF-E2	0,0308
	minSum (intermediate)	PF0014_TGACGTCA	4	**FOS/AP1**	0,0383
		PF0032_TGASTMAGC	3	NF-E2	0,0272
TRANSFAC 7.0	minFN (low)	M00055_V$NMYC_01	34	NMYC	0,0174
		M00244_V$NGFIC_01	5	NGFIC	0,0425
		M00246_V$EGR2_01	5	EGR2	0,0499
		M00251_V$XBP1_01	19	XBP1	0,02
	minFP (high)	M00185_V$NFY_Q6	6	**NFY**	0,0284
		M00188_V$AP1_Q4	7	**AP1**	0,0096
	minSum (intermediate)	M00040_V$CREBP1_01	6	**CREBP1**	0,0465
		M00185_V$NFY_Q6	14	**NFY**	0,0244
		M00244_V$NGFIC_01	5	NGFIC	0,0437
		M00287_V$NFY_01	14	**NFY**	0,0248
		M00394_V$MSX1_01	16	MSX1	0,0229

Enriched sequence motifs in core HIF binding regions, as indicated by statistical analysis (Fisher’s exact test, p<0.05 with no correction for multiple comparisons). For each overrepresented sequence motif/PWM, the table indicates the following: the database collection (PWM collection), the stringency used in *in silico* TFBS identification (Stringency), the number of hits obtained in the set of core HBRs (Hits), the transcription factor (Tr. Factor) associated to the PWM and the p value of the enrichment (p value). Robust predictions across different stringencies and PWM datasets are highlighted in bold.

As an independent assessment of enriched motifs that is less dependent on the composition of the core set, we compared the results of the previous analysis with a variable selection approach implemented in the Weka machine learning software [53]. Correlation-based feature selection was applied to the complete set of high-stringency predictions to detect non-redundant variables (PWMs) able to distinguish between the core and background sets. As expected, a number of the top-ranked PWMs, such as those for HIF1, AP1/ATF3 or NFY were coincident with the Fisher’s exact test predictions (Table 2). However, additional enriched motifs were found (such as CEBPB or NFAT), probably reflecting an increased predictive power after stratified cross-validation.

**Table 2:** Enriched TFBSs in core HIF binding regions (variable selection).

Overrepresented PWM	Number chosen	Transcription factor
MA0259.1	100	**HIF1**
PF0146	86	unknown (RRCCGTTA)
PF0032	82	NFE-2
M00222	80	Hand1:E47
M00188	77	**AP1**
PF0096	67	unknown (YGCANTGCR)
PF0014	58	**ATF3**
M00109	57	CEBPB
M00185	45	**NFY**
M00246	36	EGR2
M00244	20	NGFIC
MA0154.1	15	EBF1
PF0009	13	**ATF3**
M00302	11	NFAT
M00002	10	E47

The Table indicates sequence motifs/PWMs identified as discriminative of core HBRs employing correlation-based feature selection in 10 iterations of 10-fold stratified cross-validation. The results are ranked according to the total number of folds (up to a hundred) in which the variable was chosen by the algorithm (Number chosen). The associated transcription factor, were known, is indicated along with the PWM (Tr. factor). Predictions coincident with Fisher’s exact test (Table 1) are highlighted in bold.

We next asked whether the TFs associated to the enriched TFBSs may share any common characteristics. Gene annotation enrichment analysis (Table S7) of these enriched transcription factors pointed at stimulus-responsive transcription factors as significantly enriched in core HIF binding regions, and indeed most of the identified DNA-binding proteins have been reported to function as transcription factors of stress responses [60], including hypoxia-responsive TFs [61]. On the whole, our results suggest that binding sequences of several additional TFs other than HIFs, and in particular diverse stress-responsive TFs, are enriched in *bona fide* HIF binding regions.

### 4. Functional Impact of Transcription Factor Binding Sites Proximal to Hypoxia Response Elements

**Figure 4:**
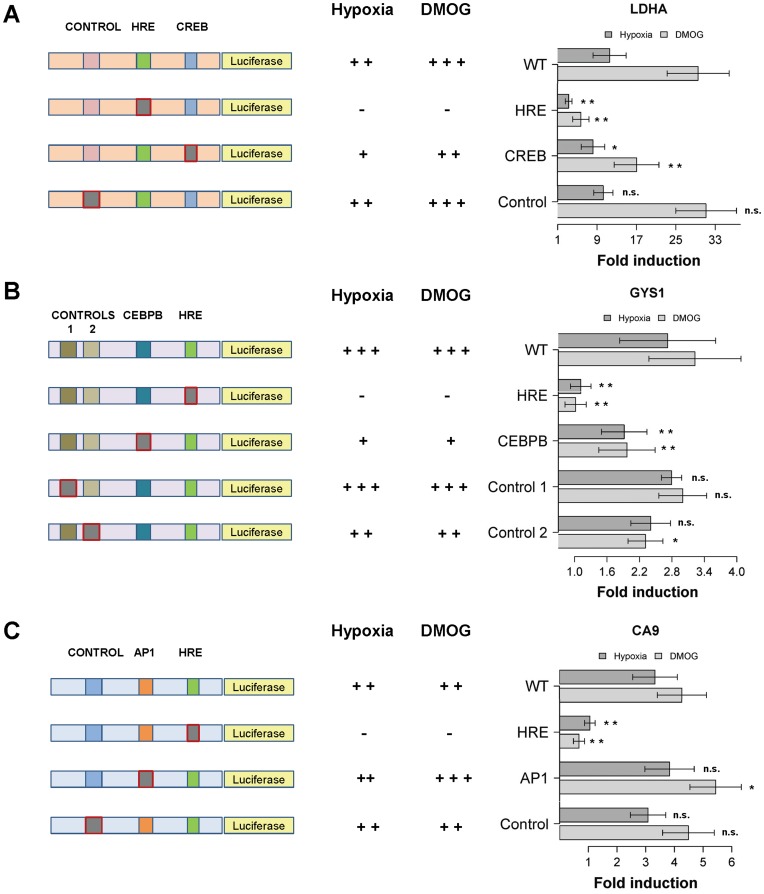
Effect of stress-responsive transcription factor binding sites in the proximity of hypoxia-response elements on hypoxic induction of HIF-responsive promoters. HeLa cells were transfected with reporter plasmids containing promoter regions of mouse LDHA (**A**), human GYS1 (**B**) and human CA9 (**C**) in their wild-type form or harbouring the indicated mutations. Diagrams to the left of each graph indicate the location of the different mutations in the employed promoter constructs (grey blocks, highlighted with red border). Effects on reporter induction by hypoxia and the hypoxia mimetic DMOG are summarized in the central columns: -, no hypoxic/DMOG induction; +, ++, +++: increasing strength of hypoxic/DMOG induction. Graphs represent the fold induction over normoxia of the wild-type promoter (WT) upon hypoxia or DMOG treatment, compared to that of promoter versions harbouring mutations in the hypoxia response element (HRE), in CREB (**A**), CEBPB (**B**), or AP1 (**C**) binding sites proximal to the HRE, or in control genomic regions (CONTROL). Bars represent average values in four to six independent experiments, and error bars the standard deviation. Statistical significance of observed activity compared to the wild-type promoters are indicated: n.s.: not significant, *: p<0.05, **: p<0.01 (repeated measures ANOVA with Dunnet post-hoc correction).

In order to address the functional relevance of the enriched TFBSs identified *in silico*, we next set out to validate some of these predictions experimentally. To this end, we selected hits for enriched TFBSs focusing on: 1) HREs located close to the TSSs of genes, to be able to study these promoter regions in *in cellulo* reporter assays, and 2) TFBS predictions located close to the Hypoxia Response Element (HRE). According to these criteria, we evaluated a CREB binding site prediction in the *LDHA* promoter (Figure 4A), a CEBPB binding site identified *in silico* in the *GYS1* promoter (Figure 4B), and a predicted AP1 site in the *CA9* promoter (Figure 4C). The selected promoters were cloned upstream of a firefly luciferase gene, either in their wild-type version or harbouring mutations in the predicted TFBSs. We then compared the effect of these mutations with that of the hypoxia response element (HRE). Finally, and in order to evaluate non-specific effects of the introduced changes, we also generated control mutations in these promoters by altering randomly-selected DNA motifs in the vicinity of the HRE (Figure 4). These control mutations lay in non-conserved (LDHA and GYS1 CONTROL 2) as well as conserved (CA9 and GYS1 CONTROL 1) genomic regions.

We next measured the luciferase activity of these constructs in normoxia, hypoxia (1% oxygen) and upon treatment with the prolyl hydroxylase inhibitor DMOG (500 µM). As expected, mutation of the HRE in all the studied promoters completely abrogated induction by either hypoxia or DMOG treatment (Figure 4). Importantly, mutation of the predicted CREB site in the proximity of the HRE within the *LDHA* promoter led to a partial reduction in the inducibility of the construct, while introduction of a random mutation had a negligible effect in the response of the promoter to either hypoxia or DMOG. Similarly, mutation of the CEBPB binding site proximal to the *GYS1* HRE led to a partial abrogation of the hypoxic induction when compared to mutation of the HRE core (Figure 4B). This reduction was not consistently recapitulated when two distinct control mutations were introduced in the promoter (Figure 4B, Control 1 and Control 2), strongly suggesting that it is a specific effect. Importantly, similar results were obtained upon DMOG treatment (Figure 4B). Finally, in contrast to the two previous cases, directed mutagenesis of the AP1 site proximal to the *CA9* HRE led to slightly increased inducibility of the construct by either hypoxia or DMOG (Figure 4C), reaching statistical significance only for the latter. This effect was distinct from that of a control mutation or the expected abrogation of the induction produced by mutation of the HRE.

Collectively, these results indicate that at least some of the TFBSs computationally predicted as enriched in a core set of *bona fide* HIF binding regions play a functional role in the transactivation by hypoxia or DMOG treatment of HIF-responsive promoters. Moreover, our data also suggests that diverse stress-responsive transcription factors, probably through modulation of basal transcription or recruitment of common cofactors, contribute to the specification of HIF target selectivity.

### 5. Transcriptional Targets of Stress-responsive Transcription Factors are Enriched Among HIF Target Genes

The results in the previous section were restricted to a limited set of validated promoters. However, if the involved transcription factors are of general relevance to HIF mediated transcription, some of their targets would be expected to be common with HIFs. To judge the potential generality of the involvement of CEBPs, CREB and AP1 in modulation induction of HIF transcriptional targets, we employed a gene-set enrichment analysis (GSEA) [55] as an unbiased way to explore the distribution of other transcription factor targets among hypoxia inducible genes. For this analysis, we employed a list of over 11000 genes sorted according to their response to hypoxia, and derived from our previous meta-analysis of gene expression profiles [13]. This sorted list was then queried against the curated collection (C2) of the GSEA molecular signatures database (http://www.broadinstitute.org/gsea/index.jsp). This collection comprises over 3000 gene sets from various sources including experimentally derived lists of targets for specific transcription factors. Thus, this analysis identifies sets of functionally related genes, such as those co-regulated in response to specific genetic and chemical perturbations, that are significantly enriched in the top positions of a list of genes induced by hypoxia.

**Figure 5:**
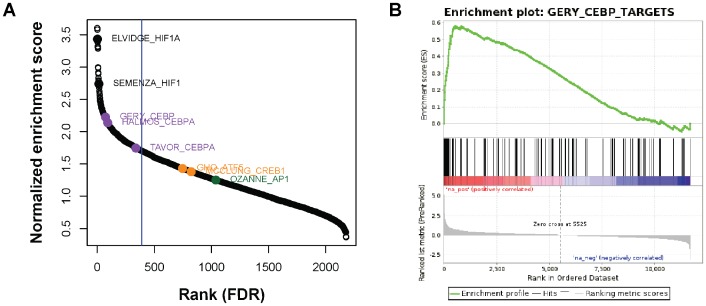
Transcriptional targets of stress-responsive transcription factors are enriched among core hypoxia-responsive genes. (**A**) Gene-set enrichment analysis on a set of 11673 genes sorted by their response to hypoxia according to a meta-analysis of hypoxia gene expression experiments (ref. 13). The graph depicts the normalized enrichment score of 3174 gene sets from the curated collection (C2) of the GSEA molecular signatures database v3.0, that includes sets of transcription factor target genes. Solid circles highlight gene-sets derived from studies on HIF1 (black, ELVIDGE_HYPOXIA_UP and SEMENZA_HIF1_TARGETS), CEBPA/B (purple, GERY_CEBP_TARGETS, HALMOS_CEBPA_TARGETS_UP and TAVOR_CEBPA_TARGETS_UP), CREB1/ATF5 (orange, GHO_ATF5_TARGETS_DN and MCCLUNG_CREB1_TARGETS_UP) and AP1 (green, OZANNE_AP1_TARGETS_UP) transcriptional targets. The vertical blue line corresponds to an FDR-adjusted p-value of 0.05. (**B**) GSEA analysis of hypoxia-responsive genes (see A) against the GERY_CEBPA_TARGETS (M12338, derived from the GEO dataset GSE2188) gene-set. Hypoxic response is rank-ordered in the horizontal axis (Rank in ordered dataset). Black bars indicate the position of individual targets in the CEBPA gene-set. The graph on top (green curve) represents enrichment scores of CEBPA targets across hypoxia responsive genes, indicating positive correlation between the two. The gradient color bar indicates positive (red) and negative (blue) correlation boundaries.

As expected, GSEA analysis revealed a statistically significant enrichment of well-characterized HIF targets in this sorted list (Figure 5A, black circles). Moreover, enrichment of CEBPA/B targets was also significant for three different gene-sets (Figure 5A, purple circles, and Figure 5B). These gene sets derive from independent expression profiling experiments performed in cells overexpressing different members of the CEBP family [62]–[64]. In addition, the analysis also revealed enrichment for two gene sets containing targets regulated by the ATF/CREB family (Figure 5A, orange circles), albeit the FDR-adjusted p-values did not reach statistical significance (0.106 and 0.229 respectively). Finally, enrichment of AP1 targets was not statistically significant (Figure 5A, green circle). Altogether, these results suggest, at least for the case of CEBPs (Figure 5B), that transcription factor collaboration can be a general mechanism contributing to HIF target selectivity.

## Discussion

The complete elucidation of the molecular principles governing the translation of genomic information to gene regulation remains a central question in biology. In particular, understanding the mechanisms dictating target selection by HIF transcription factors is of fundamental importance to truly dissect the genes directly modulated by HIFs, and therefore to completely characterize the transcriptional response to hypoxia that these factors orchestrate, and its interactions with other transcriptional pathways. Several mechanisms have been proposed to contribute to selective DNA binding and gene regulation by transcription factors with largely generic DNA binding domains [65], among them the co-binding of several transcription factor molecules [14], [66], [67]. In order to dissect these mechanisms, high-quality collections of binding sites are an obvious pre-requisite. The recent development of high-throughput chromatin immunoprecipitation experiments [41], [68] has spurred knowledge on the genome-wide DNA binding locations of transcription factors, and these techniques hence constitute an essential tool to explore mechanisms of transcriptional regulation on a global scale [69]–[71]. In this work, we employed an integrative approach to identify additional transcription factors that could contribute to HIFs binding and target selectivity. This strategy was based on computational prediction of enriched sequence motifs in a set of core HIF binding regions constructed through selection of HIF1 alpha binding locations derived from genome-wide chromatin immunoprecipitation experiments in HeLa (this study), HepG2 [9], MCF-7 [10] and U87 cells [11]. During preparation of this manuscript, a fourth study employing ChIP-Seq in MCF-7 cells was published [12], providing high-resolution data on genome-wide HIF binding locations independently of gene architecture.

Chromatin accessibility has been shown to play an important role in dictating transcription factor binding [72]–[74]. In this regard, integration of HIF1 alpha binding locations in U87 and HepG2 cells with gene expression data in the same cell types revealed a preference for HIF1 binding to map to transcriptionally active genes in normoxia [11], therefore suggesting that chromatin accessibility, as indirectly evidenced by basal transcriptional activity, determines HIF1 binding. As an independent approach to test this hypothesis, we looked at the correlation of normoxic gene expression and induction of known HIF targets in publicly available microarray datasets of hypoxic cell cultures [13]. In agreement, we found a statistically significant association between basal expression and hypoxia inducibility of known targets (Figure 1A and Table S2). Furthermore, comparison of HIF1α and HIF2α binding locations in MCF-7 cells with DNAse hypersentitivity data in the same cell type [12] also revealed a significant association of HIF binding with normoxic DNAse hypersensitive sites, again pointing at an important role of open chromatin regions in dicating HIF binding. However, when conserved RCGTG HIF binding consensus motifs are identified in non-coding regions of genes showing basal expression, a majority of these are not induced by hypoxia (Figure 3B). Therefore, although chromatin accessibility clearly favors HIF1 binding, additional mechanisms are likely needed to fully specify HIF target selectivity.

DNA methylation of a HIF binding site was originally shown to block HIF1α binding to the 3′ erythropoietin enhancer [16], and indeed erythropoietin expression appears to be restricted to cell types in which the hypoxia response element is unmethylated. Altered HIF binding due to methylation changes in HREs has been further validated in additional target genes, such as *BNIP3* [75] or *HIF1A* [76], and is often associated with cancer progression. However, a global view on the effects of DNA methylation in HIF binding selectivity is lacking, and may be challenging to analyze in view of recent evidence arguing for dynamic DNA methylation in hypoxia [77].

Additional transcription factors binding in the proximity of a HIF1 binding site could impact either HIF1 binding or transcriptional modulation of the target gene. In agreement with this possibility, a recent study addressing the functional validation of common genetic variants at a renal cancer susceptibility locus [78] found HIF2 binding to be dependent on a polymorphism falling outside the RCGTG HIF binding consensus, strongly suggesting that sequences outside the HIF binding site can be functionally important in determining HIF binding. We tested this hypothesis by computational prediction of transcription factor binding sites enriched in a core set of *bona fide* HIF binding regions (Figure 3B). These were obtained through integration of HIF1α ChIP-chip data with a gene expression meta-analysis of hypoxic cell cultures [13] (Figure 3A), thereby combining multiple HIF DNA binding and hypoxic gene expression datasets. Our approach has the advantage of using an integrated set of sequences that could overcome the limitations of analyses based on a single dataset [10], [30], where a proportion of binding sites could potentially correspond to false positives or non-functional sites. In addition to HIF matrices, we observed additional sequence motifs that were enriched in core HIF binding regions (Tables 1 and 2) and that could potentially impact HIF binding and transactivation selectivity. Of note, the transcriptional activity of several of these proteins, such as AP-1, CREB, EGR-2 or CEBPB is known to be induced by hypoxia [61]. Nevertheless, and in agreement with previous predictions of enriched TFBSs in the vicinity of experimentally [10] or computationally [30] identified HIF binding sites, the statistical significance of these predictions is relatively low and, even on an integrated dataset, no single collaborating TF stands out. In fact, HIF PWMs are the most enriched in core HIF binding regions. Since sequences in the background set used for comparison also contain RCGTG motifs (Figure S1), this enrichment likely arises from the well known preference for A versus G in the first position of the HIF binding consensus. These results collectively suggest that several additional transcription factors could influence HIF transcriptional activity.

Importantly, we noted that most of the enriched TFBSs corresponded to stress-responsive transcription factors. Varied stress-responsive TFs have been shown to coordinately regulate the same genes [23], [79], and indeed several transcription factors are activated by the same stresses in mammalian cells [61], [80], [81]. However, it is unclear whether this cooperation among stress-responsive pathways translates at the genomic level. In order to evaluate the functional significance of the TFBSs enriched in core HIF binding regions, we carried out an experimental validation by disrupting selected sequences in *bona fide* HIF-responsive promoters (Figure 4). Importantly, no experimental confirmation had been attempted on previously reported predictions [10], [30], and therefore the biological significance of those findings remained unclear. In spite of being limited to three selected promoters, our results clearly indicate that, compared to control mutations, alteration of binding sequences of transcription factors enriched in HIF binding regions, and different from HIFs themselves, have a specific effect on the transcriptional activation of HIF-responsive promoters. In particular, we found negative effects on hypoxic induction of *LDHA* and *GYS1* promoters upon disruption of CREB and CEBPB binding sites proximal to the HRE, whereas mutation of an AP1 site proximal to the CA9 HRE led to a slightly augmented hypoxic induction of the promoter. In agreement with our results, mutation of the same CREB binding site was been previously shown to alter *LDHA* hypoxic induction [26]. Interestingly, USF binding to a palindrome CACGTG HRE in the *LDHA* promoter was suggested to complement HIF binding [27]. However, our results do not allow us to corroborate these findings, as mutation of this HRE was not evaluated in our experiments (Materials and Methods and Table S1). Furthermore, hypoxic *CA9* expression has been linked to cooperation between AP1 family member ATF4 and HIF1α [82]. In this study, ATF4 overexpression led to an augmented *CA9* induction in hypoxia, with reduced hypoxic expression of *CA9* being observed upon ATF4 knock-down. Chromatin immunoprecipitation experiments mapped ATF4 binding to the −1400/−1000 region of the *CA9* promoter, which falls outside of the promoter region employed in our experiments. Nevertheless, the apparent paradox with our results argues for careful interpretation of the role of AP1 in the HIF transcriptional response. In fact, both positive and negative effects of AP1 have been reported on hypoxic gene expression [81] and, given the number of AP1 family members, these probably arise from compositional differences in AP1 complexes.

Importantly, the effects observed upon mutation of CREB, CEBPB or AP1 binding sites (Figure 4) were always moderate when compared to mutation of the HIF binding consensus RCGTG, suggesting that rather than being an absolute requirement for hypoxic induction, the integrity of these neighboring TFBSs fine-tunes the HIF-mediated transcriptional response. Thus, it is possible that multiple independent factors contribute, in an additive fashion, to HIF-mediated transcription. This model could also explain why we found a relatively large number of enriched TFBSs in HIF binding regions, but all of them sharing a modest statistical significance. On the whole, these observations indicate that several of the enriched TFBSs identified in our approach are of functional relevance for HIF-mediated transcription. Nevertheless, it should be noted that other TFs for which collaboration with HIFs has been previously suggested [61] are not recovered as enriched in our approach. An inherent assumption in our method is that such TFBSs will be enriched in bona fide HIF binding regions (Figure 3), which may not hold true if cooperation is specific to a small number of target genes. Furthermore, the employed HIF binding data in this study is for the HIF1α subunit only, whereas transcription factor cooperativity may well apply to other HIF subunits. In fact, several reports have implicated the ETS family of transcription factors in target selection by HIF2α [28], [29].

We observed very similar tendencies when transcriptional activation of reporter constructs was elicited by DMOG or hypoxia treatment (Figure 4), additionally suggesting that, at least in our experimental conditions, the contribution of these factors could occur mainly in basal conditions, as it is unlikely that hypoxia and DMOG treatment induce completely overlapping cellular responses. Several recent reports [11], [12] have suggested that chromatin accessibility determines HIF1 binding, although this mechanism may not fully explain HIFs binding and target selectivity (Figure 1). Our results indicate that an additional layer of specificity comes from proximal co-binding of other transcription factors and HIFs to open chromatin regions, thereby facilitating or restricting HIF-mediated transcription. Elucidation of the underlying molecular mechanisms falls outside the scope of our work, although it is tempting to speculate that transcription factors binding in proximity of HIFs may be involved in recruitment of co-activator or co-repressor proteins. Of note, a recent mammalian two-hybrid survey of protein-protein interactions for human and mouse TFs [83] reported a physical association between HIF1A and AP-1 family member JUN, as well as the previously known interaction between CEBPB and p300 [84]. p300/CBP is a master co-activator of HIF-mediated transcription whose recruitment can also be mediated by CREB [85]. In this regard, evidence from a synthetic transactivation screen on the EGLN1 promoter [86] pointed to ETV4 as an additional p300-dependent coactivator of HIF-mediated transcription. Moreover, HIF1 is known to interact with Jab1/CSN5 [87], [88], a protein originally identified as a transcriptional co-activator for AP1 [89]. Future investigations on protein composition of HIF-bound enhancers should be pivotal in supporting this model.

The associations between HIFs and AP1, CREB and CEBPs analyzed in our reporter results could be general across many HIF targets or be restricted to individual targets. To judge the generality of these results, we conducted a gene-set enrichment analysis (GSEA) of transcription factor targets in a sorted list of genes regulated by hypoxia [13]. The results of this analysis showed a significant enrichment of CEBP targets among hypoxia-inducible genes (Figure 5), suggesting that at least for this family of transcription factors, the functional association with HIFs could be relatively general. Of note, recent works have reported a direct protein-protein interaction between HIF1α and CEBPα [90], [91], and have implicated CEBPα activity in regulation of the HIF target genes galectin-1 [92] and PAI-1 [23]. Hypoxic induction of both *galectin-1* and *PAI-1* was found to be synergistically dependent on both HIF1α and CEBPα activity and their co-binding to the promoter region. Our results further suggest that this functional association may be general across a wider collection of HIF targets.

In conclusion, the data presented herein demonstrates that integration of high-throughput chromatin immunoprecipitation and gene expression data is a successful approach to select high-quality core HIF binding regions, and provides experimental proof of principle for the biological relevance of enriched transcription factor binding sites other than the HIF binding consensus in HIF-mediated transcription. Specifically, our results suggest that diverse stress-responsive transcription factors, in particular CEBPs, contribute to fine-tuning of the HIF-mediated transcriptional response.

## Supporting Information

Figure S1 **Construction of a background set of control sequences resembling core HIF binding regions.**
(PDF)

Table S1 **HRE, AP1, CREB, CEBPB binding sites and control mutations introduced in LDHA, CA9 and GYS1 promoter constructs.**
(XLSX)

Table S2 **Correlation between normoxic basal expression and hypoxia inducibility (known HIF targets).**
(XLSX)

Table S3 **Candidate HIF1alpha-binding regions identified by ChIP-chip analysis in HeLa cells.**
(XLSX)

Table S4 **Distribution of conserved HBSs in ChIP-chip results across different cell types.**
(XLSX)

Table S5 **Integrated set of core HIF binding regions.**
(XLSX)

Table S6 **Enriched gene annotation clusters in core HIF binding regions.**
(XLSX)

Table S7 **Enriched gene annotation terms in transcription factors associated to sequence motifs overrepresented in core HIF binding regions.**
(XLSX)

## References

[1] Wang GL, Jiang BH, Rue EA, Semenza GL (1995) Hypoxia-inducible factor 1 is a basic-helix-loop-helix-PAS heterodimer regulated by cellular O2 tension. Proc Natl Acad Sci U S A 92: 5510–5514. 7539918 10.1073/pnas.92.12.5510 PMC41725

[2] Salceda S, Caro J (1997) Hypoxia-inducible factor 1alpha (HIF-1alpha) protein is rapidly degraded by the ubiquitin-proteasome system under normoxic conditions. Its stabilization by hypoxia depends on redox-induced changes. J Biol Chem 272: 22642–22647. 9278421 10.1074/jbc.272.36.22642

[3] Mahon PC, Hirota K, Semenza GL (2001) FIH-1: a novel protein that interacts with HIF-1alpha and VHL to mediate repression of HIF-1 transcriptional activity. Genes Dev 15: 2675–2686. 11641274 10.1101/gad.924501 PMC312814

[4] Epstein AC, Gleadle JM, McNeill LA, Hewitson KS, O’Rourke J, et al (2001) C. elegans EGL-9 and mammalian homologs define a family of dioxygenases that regulate HIF by prolyl hydroxylation. Cell 107: 43–54. 11595184 10.1016/s0092-8674(01)00507-4

[5] Bruick RK, McKnight SL (2001) A conserved family of prolyl-4-hydroxylases that modify HIF. Science 294: 1337–1340. 11598268 10.1126/science.1066373

[6] Lando D, Peet DJ, Whelan DA, Gorman JJ, Whitelaw ML (2002) Asparagine hydroxylation of the HIF transactivation domain a hypoxic switch. Science 295: 858–861. 11823643 10.1126/science.1068592

[7] Wenger RH, Stiehl DP, Camenisch G (2005) Integration of oxygen signaling at the consensus HRE. Sci STKE 2005: re12. 16234508 10.1126/stke.3062005re12

[8] Kaelin WG Jr, Ratcliffe PJ (2008) Oxygen sensing by metazoans: the central role of the HIF hydroxylase pathway. Mol Cell 30: 393–402. 18498744 10.1016/j.molcel.2008.04.009

[9] Xia X, Lemieux ME, Li W, Carroll JS, Brown M, et al (2009) Integrative analysis of HIF binding and transactivation reveals its role in maintaining histone methylation homeostasis. Proc Natl Acad Sci U S A 106: 4260–4265. 19255431 10.1073/pnas.0810067106 PMC2657396

[10] Mole DR, Blancher C, Copley RR, Pollard PJ, Gleadle JM, et al (2009) Genome-wide association of hypoxia-inducible factor (HIF)-1{alpha} and HIF-2{alpha} DNA binding with expression profiling of hypoxia-inducible transcripts. J Biol Chem 284: 16767–16775. 19386601 10.1074/jbc.M901790200 PMC2719312

[11] Xia X, Kung AL (2009) Preferential binding of HIF-1 to transcriptionally active loci determines cell-type specific response to hypoxia. Genome Biol 10: R113. 19828020 10.1186/gb-2009-10-10-r113 PMC2784328

[12] Schodel J, Oikonomopoulos S, Ragoussis J, Pugh CW, Ratcliffe PJ, et al (2011) High-resolution genome-wide mapping of HIF-binding sites by ChIP-seq. Blood 117: e207–217. 21447827 10.1182/blood-2010-10-314427 PMC3374576

[13] Ortiz-Barahona A, Villar D, Pescador N, Amigo J, del Peso L (2010) Genome-wide identification of hypoxia-inducible factor binding sites and target genes by a probabilistic model integrating transcription-profiling data and in silico binding site prediction. Nucleic Acids Res 38: 2332–2345. 20061373 10.1093/nar/gkp1205 PMC2853119

[14] Pan Y, Tsai CJ, Ma B, Nussinov R (2010) Mechanisms of transcription factor selectivity. Trends Genet 26: 75–83. 20074831 10.1016/j.tig.2009.12.003 PMC7316385

[15] Bell O, Tiwari VK, Thoma NH, Schubeler D (2011) Determinants and dynamics of genome accessibility. Nat Rev Genet 12: 554–564. 21747402 10.1038/nrg3017

[16] Wenger RH, Kvietikova I, Rolfs A, Camenisch G, Gassmann M (1998) Oxygen-regulated erythropoietin gene expression is dependent on a CpG methylation-free hypoxia-inducible factor-1 DNA-binding site. Eur J Biochem 253: 771–777. 9654078 10.1046/j.1432-1327.1998.2530771.x

[17] Rossler J, Stolze I, Frede S, Freitag P, Schweigerer L, et al (2004) Hypoxia-induced erythropoietin expression in human neuroblastoma requires a methylation free HIF-1 binding site. J Cell Biochem 93: 153–161. 15352172 10.1002/jcb.20133

[18] Panne D (2008) The enhanceosome. Curr Opin Struct Biol 18: 236–242. 18206362 10.1016/j.sbi.2007.12.002

[19] Kulkarni MM, Arnosti DN (2003) Information display by transcriptional enhancers. Development 130: 6569–6575. 14660545 10.1242/dev.00890

[20] Segal E, Widom J (2009) From DNA sequence to transcriptional behaviour: a quantitative approach. Nat Rev Genet 10: 443–456. 19506578 10.1038/nrg2591 PMC2719885

[21] Zhang W, Tsuchiya T, Yasukochi Y (1999) Transitional change in interaction between HIF-1 and HNF-4 in response to hypoxia. J Hum Genet 44: 293–299. 10496070 10.1007/s100380050163

[22] Yamashita K, Discher DJ, Hu J, Bishopric NH, Webster KA (2001) Molecular regulation of the endothelin-1 gene by hypoxia. Contributions of hypoxia-inducible factor-1, activator protein-1, GATA-2, AND p300/CBP. J Biol Chem 276: 12645–12653. 11278891 10.1074/jbc.M011344200

[23] Liao H, Hyman MC, Lawrence DA, Pinsky DJ (2007) Molecular regulation of the PAI-1 gene by hypoxia: contributions of Egr-1, HIF-1alpha, and C/EBPalpha. Faseb J 21: 935–949. 17197388 10.1096/fj.06-6285com

[24] Sanchez-Elsner T, Ramirez JR, Sanz-Rodriguez F, Varela E, Bernabeu C, et al (2004) A cross-talk between hypoxia and TGF-beta orchestrates erythropoietin gene regulation through SP1 and Smads. J Mol Biol 336: 9–24. 14741200 10.1016/j.jmb.2003.12.023

[25] Miki N, Ikuta M, Matsui T (2004) Hypoxia-induced activation of the retinoic acid receptor-related orphan receptor alpha4 gene by an interaction between hypoxia-inducible factor-1 and Sp1. J Biol Chem 279: 15025–15031. 14742449 10.1074/jbc.M313186200

[26] Firth JD, Ebert BL, Ratcliffe PJ (1995) Hypoxic regulation of lactate dehydrogenase A. Interaction between hypoxia-inducible factor 1 and cAMP response elements. J Biol Chem 270: 21021–21027. 7673128 10.1074/jbc.270.36.21021

[27] Hu J, Stiehl DP, Setzer C, Wichmann D, Shinde DA, et al (2011) Interaction of HIF and USF signaling pathways in human genes flanked by hypoxia-response elements and E-box palindromes. Mol Cancer Res 9: 1520–1536. 21984181 10.1158/1541-7786.MCR-11-0090

[28] Aprelikova O, Wood M, Tackett S, Chandramouli GV, Barrett JC (2006) Role of ETS transcription factors in the hypoxia-inducible factor-2 target gene selection. Cancer Res 66: 5641–5647. 16740701 10.1158/0008-5472.CAN-05-3345

[29] Hu CJ, Sataur A, Wang L, Chen H, Simon MC (2007) The N-terminal transactivation domain confers target gene specificity of hypoxia-inducible factors HIF-1alpha and HIF-2alpha. Mol Biol Cell 18: 4528–4542. 17804822 10.1091/mbc.E06-05-0419 PMC2043574

[30] Benita Y, Kikuchi H, Smith AD, Zhang MQ, Chung DC, et al (2009) An integrative genomics approach identifies Hypoxia Inducible Factor-1 (HIF-1)-target genes that form the core response to hypoxia. Nucleic Acids Res 37: 4587–4602. 19491311 10.1093/nar/gkp425 PMC2724271

[31] Hu CJ, Wang LY, Chodosh LA, Keith B, Simon MC (2003) Differential roles of hypoxia-inducible factor 1alpha (HIF-1alpha) and HIF-2alpha in hypoxic gene regulation. Mol Cell Biol 23: 9361–9374. 14645546 10.1128/MCB.23.24.9361-9374.2003 PMC309606

[32] Vengellur A, Woods BG, Ryan HE, Johnson RS, LaPres JJ (2003) Gene expression profiling of the hypoxia signaling pathway in hypoxia-inducible factor 1alpha null mouse embryonic fibroblasts. Gene Expr 11: 181–197. 14686790 10.3727/000000003108749062 PMC5991159

[33] Greijer AE, van der Groep P, Kemming D, Shvarts A, Semenza GL, et al (2005) Up-regulation of gene expression by hypoxia is mediated predominantly by hypoxia-inducible factor 1 (HIF-1). J Pathol 206: 291–304. 15906272 10.1002/path.1778

[34] Chi JT, Wang Z, Nuyten DS, Rodriguez EH, Schaner ME, et al (2006) Gene expression programs in response to hypoxia: cell type specificity and prognostic significance in human cancers. PLoS Med 3: e47. 16417408 10.1371/journal.pmed.0030047 PMC1334226

[35] Vengellur A, Phillips JM, Hogenesch JB, LaPres JJ (2005) Gene expression profiling of hypoxia signaling in human hepatocellular carcinoma cells. Physiol Genomics 22: 308–318. 15942021 10.1152/physiolgenomics.00045.2004

[36] Elvidge GP, Glenny L, Appelhoff RJ, Ratcliffe PJ, Ragoussis J, et al (2006) Concordant regulation of gene expression by hypoxia and 2-oxoglutarate-dependent dioxygenase inhibition: the role of HIF-1alpha, HIF-2alpha, and other pathways. J Biol Chem 281: 15215–15226. 16565084 10.1074/jbc.M511408200

[37] Katada K, Naito Y, Mizushima K, Takagi T, Handa O, et al (2006) Gene expression profiles on hypoxia and reoxygenation in rat gastric epithelial cells: a high-density DNA microarray analysis. Digestion 73: 89–100. 16788290 10.1159/000094039

[38] Sung FL, Hui EP, Tao Q, Li H, Tsui NB, et al (2007) Genome-wide expression analysis using microarray identified complex signaling pathways modulated by hypoxia in nasopharyngeal carcinoma. Cancer Lett 253: 74–88. 17320280 10.1016/j.canlet.2007.01.012

[39] Edgar R, Domrachev M, Lash AE (2002) Gene Expression Omnibus: NCBI gene expression and hybridization array data repository. Nucleic Acids Res 30: 207–210. 11752295 10.1093/nar/30.1.207 PMC99122

[40] Pescador N, Cuevas Y, Naranjo S, Alcaide M, Villar D, et al (2005) Identification of a functional hypoxia-responsive element that regulates the expression of the egl nine homologue 3 (egln3/phd3) gene. Biochem J 390: 189–197. 15823097 10.1042/BJ20042121 PMC1184574

[41] Ren B, Robert F, Wyrick JJ, Aparicio O, Jennings EG, et al (2000) Genome-wide location and function of DNA binding proteins. Science 290: 2306–2309. 11125145 10.1126/science.290.5500.2306

[42] Singer GA, Wu J, Yan P, Plass C, Huang TH, et al (2008) Genome-wide analysis of alternative promoters of human genes using a custom promoter tiling array. BMC Genomics 9: 349. 18655706 10.1186/1471-2164-9-349 PMC2527337

[43] Slater GS, Birney E (2005) Automated generation of heuristics for biological sequence comparison. BMC Bioinformatics 6: 31. 15713233 10.1186/1471-2105-6-31 PMC553969

[44] Toedling J, Skylar O, Krueger T, Fischer JJ, Sperling S, et al (2007) Ringo–an R/Bioconductor package for analyzing ChIP-chip readouts. BMC Bioinformatics 8: 221. 17594472 10.1186/1471-2105-8-221 PMC1906858

[45] Smyth GK (2005) Limma: linear models for microarray data. In: R. Gentleman V, Carey SD, R Irizarry, W Huber, editors. Bioinformatics and Computational Biology Solutions using R and Bioconductor. New York: Springer. 397–420.

[46] Toedling J, Huber W (2008) Analyzing ChIP-chip data using bioconductor. PLoS Comput Biol 4: e1000227. 19043553 10.1371/journal.pcbi.1000227 PMC2582686

[47] Siepel A, Bejerano G, Pedersen JS, Hinrichs AS, Hou M, et al (2005) Evolutionarily conserved elements in vertebrate, insect, worm, and yeast genomes. Genome Res 15: 1034–1050. 16024819 10.1101/gr.3715005 PMC1182216

[48] Rhead B, Karolchik D, Kuhn RM, Hinrichs AS, Zweig AS, et al (2010) The UCSC Genome Browser database: update 2010. Nucleic Acids Res 38: D613–619. 19906737 10.1093/nar/gkp939 PMC2808870

[49] Portales-Casamar E, Thongjuea S, Kwon AT, Arenillas D, Zhao X, et al (2010) JASPAR 2010: the greatly expanded open-access database of transcription factor binding profiles. Nucleic Acids Res 38: D105–110. 19906716 10.1093/nar/gkp950 PMC2808906

[50] Matys V, Kel-Margoulis OV, Fricke E, Liebich I, Land S, et al (2006) TRANSFAC and its module TRANSCompel: transcriptional gene regulation in eukaryotes. Nucleic Acids Res 34: D108–110. 16381825 10.1093/nar/gkj143 PMC1347505

[51] Stormo GD (2000) DNA binding sites: representation and discovery. Bioinformatics 16: 16–23. 10812473 10.1093/bioinformatics/16.1.16

[52] Kel AE, Gossling E, Reuter I, Cheremushkin E, Kel-Margoulis OV, et al (2003) MATCH: A tool for searching transcription factor binding sites in DNA sequences. Nucleic Acids Res 31: 3576–3579. 12824369 10.1093/nar/gkg585 PMC169193

[53] Frank E, Hall M, Trigg L, Holmes G, Witten IH (2004) Data mining in bioinformatics using Weka. Bioinformatics 20: 2479–2481. 15073010 10.1093/bioinformatics/bth261

[54] Pescador N, Villar D, Cifuentes D, Garcia-Rocha M, Ortiz-Barahona A, et al (2010) Hypoxia promotes glycogen accumulation through hypoxia inducible factor (HIF)-mediated induction of glycogen synthase 1. PLoS One 5: e9644. 20300197 10.1371/journal.pone.0009644 PMC2837373

[55] Subramanian A, Tamayo P, Mootha VK, Mukherjee S, Ebert BL, et al (2005) Gene set enrichment analysis: a knowledge-based approach for interpreting genome-wide expression profiles. Proc Natl Acad Sci U S A 102: 15545–15550. 16199517 10.1073/pnas.0506580102 PMC1239896

[56] Hemberg M, Kreiman G (2011) Conservation of transcription factor binding events predicts gene expression across species. Nucleic Acids Res 39: 7092–7102. 21622661 10.1093/nar/gkr404 PMC3167604

[57] He Q, Bardet AF, Patton B, Purvis J, Johnston J, et al (2011) High conservation of transcription factor binding and evidence for combinatorial regulation across six Drosophila species. Nat Genet 43: 414–420. 21478888 10.1038/ng.808

[58] Brahimi-Horn MC, Bellot G, Pouyssegur J (2011) Hypoxia and energetic tumour metabolism. Curr Opin Genet Dev 21: 67–72. 21074987 10.1016/j.gde.2010.10.006

[59] Ji H, Vokes SA, Wong WH (2006) A comparative analysis of genome-wide chromatin immunoprecipitation data for mammalian transcription factors. Nucleic Acids Res 34: e146. 17090591 10.1093/nar/gkl803 PMC1669715

[60] Tacchini L, Fusar-Poli D, Bernelli-Zazzera A (2002) Activation of transcription factors by drugs inducing oxidative stress in rat liver. Biochem Pharmacol 63: 139–148. 11841787 10.1016/s0006-2952(01)00836-x

[61] Cummins EP, Taylor CT (2005) Hypoxia-responsive transcription factors. Pflugers Arch 450: 363–371. 16007431 10.1007/s00424-005-1413-7

[62] Tavor S, Park DJ, Gery S, Vuong PT, Gombart AF, et al (2003) Restoration of C/EBPalpha expression in a BCR-ABL+ cell line induces terminal granulocytic differentiation. J Biol Chem 278: 52651–52659. 14517214 10.1074/jbc.M307077200

[63] Halmos B, Basseres DS, Monti S, D’Alo F, Dayaram T, et al (2004) A transcriptional profiling study of CCAAT/enhancer binding protein targets identifies hepatocyte nuclear factor 3 beta as a novel tumor suppressor in lung cancer. Cancer Res 64: 4137–4147. 15205324 10.1158/0008-5472.CAN-03-4052

[64] Gery S, Gombart AF, Yi WS, Koeffler C, Hofmann WK, et al (2005) Transcription profiling of C/EBP targets identifies Per2 as a gene implicated in myeloid leukemia. Blood 106: 2827–2836. 15985538 10.1182/blood-2005-01-0358 PMC1895299

[65] Georges AB, Benayoun BA, Caburet S, Veitia RA (2010) Generic binding sites, generic DNA-binding domains: where does specific promoter recognition come from? Faseb J 24: 346–356. 19762556 10.1096/fj.09-142117

[66] Remenyi A, Scholer HR, Wilmanns M (2004) Combinatorial control of gene expression. Nat Struct Mol Biol 11: 812–815. 15332082 10.1038/nsmb820

[67] Tomancak P, Ohler U (2010) Mapping the complexity of transcription control in higher eukaryotes. Genome Biol 11: 115. 20441601 10.1186/gb-2010-11-4-115 PMC2884534

[68] Robertson G, Hirst M, Bainbridge M, Bilenky M, Zhao Y, et al (2007) Genome-wide profiles of STAT1 DNA association using chromatin immunoprecipitation and massively parallel sequencing. Nat Methods 4: 651–657. 17558387 10.1038/nmeth1068

[69] Lefterova MI, Zhang Y, Steger DJ, Schupp M, Schug J, et al (2008) PPARgamma and C/EBP factors orchestrate adipocyte biology via adjacent binding on a genome-wide scale. Genes Dev 22: 2941–2952. 18981473 10.1101/gad.1709008 PMC2577797

[70] Farnham PJ (2009) Insights from genomic profiling of transcription factors. Nat Rev Genet 10: 605–616. 19668247 10.1038/nrg2636 PMC2846386

[71] Lupien M, Eeckhoute J, Meyer CA, Wang Q, Zhang Y, et al (2008) FoxA1 translates epigenetic signatures into enhancer-driven lineage-specific transcription. Cell 132: 958–970. 18358809 10.1016/j.cell.2008.01.018 PMC2323438

[72] Kaplan T, Biggin MD (2012) Quantitative models of the mechanisms that control genome-wide patterns of animal transcription factor binding. Methods Cell Biol 110: 263–283. 22482953 10.1016/B978-0-12-388403-9.00011-4

[73] Pique-Regi R, Degner JF, Pai AA, Gaffney DJ, Gilad Y, et al (2011) Accurate inference of transcription factor binding from DNA sequence and chromatin accessibility data. Genome Res 21: 447–455. 21106904 10.1101/gr.112623.110 PMC3044858

[74] Degner JF, Pai AA, Pique-Regi R, Veyrieras JB, Gaffney DJ, et al (2012) DNase I sensitivity QTLs are a major determinant of human expression variation. Nature 482: 390–394. 22307276 10.1038/nature10808 PMC3501342

[75] Bacon AL, Fox S, Turley H, Harris AL (2007) Selective silencing of the hypoxia-inducible factor 1 target gene BNIP3 by histone deacetylation and methylation in colorectal cancer. Oncogene 26: 132–141. 16799636 10.1038/sj.onc.1209761

[76] Koslowski M, Luxemburger U, Tureci O, Sahin U (2011) Tumor-associated CpG demethylation augments hypoxia-induced effects by positive autoregulation of HIF-1alpha. Oncogene 30: 876–882. 21042279 10.1038/onc.2010.481

[77] Liu Q, Liu L, Zhao Y, Zhang J, Wang D, et al (2011) Hypoxia induces genomic DNA demethylation through the activation of HIF-1alpha and transcriptional upregulation of MAT2A in hepatoma cells. Mol Cancer Ther 10: 1113–1123. 21460102 10.1158/1535-7163.MCT-10-1010

[78] Schodel J, Bardella C, Sciesielski LK, Brown JM, Pugh CW, et al.. (2012) Common genetic variants at the 11q13.3 renal cancer susceptibility locus influence binding of HIF to an enhancer of cyclin D1 expression. Nat Genet 44: 420–425, S421–422. 10.1038/ng.2204 PMC3378637 22406644

[79] Alam J, Cook JL (2007) How many transcription factors does it take to turn on the heme oxygenase-1 gene? Am J Respir Cell Mol Biol 36: 166–174. 16990612 10.1165/rcmb.2006-0340TR

[80] Malhi H, Kaufman RJ (2011) Endoplasmic reticulum stress in liver disease. J Hepatol 54: 795–809. 21145844 10.1016/j.jhep.2010.11.005 PMC3375108

[81] Laderoute KR (2005) The interaction between HIF-1 and AP-1 transcription factors in response to low oxygen. Semin Cell Dev Biol 16: 502–513. 16144688 10.1016/j.semcdb.2005.03.005

[82] van den Beucken T, Koritzinsky M, Niessen H, Dubois L, Savelkouls K, et al (2009) Hypoxia-induced expression of carbonic anhydrase 9 is dependent on the unfolded protein response. J Biol Chem 284: 24204–24212. 19564335 10.1074/jbc.M109.006510 PMC2782014

[83] Ravasi T, Suzuki H, Cannistraci CV, Katayama S, Bajic VB, et al (2010) An atlas of combinatorial transcriptional regulation in mouse and man. Cell 140: 744–752. 20211142 10.1016/j.cell.2010.01.044 PMC2836267

[84] Kovacs KA, Steinmann M, Magistretti PJ, Halfon O, Cardinaux JR (2003) CCAAT/enhancer-binding protein family members recruit the coactivator CREB-binding protein and trigger its phosphorylation. J Biol Chem 278: 36959–36965. 12857754 10.1074/jbc.M303147200

[85] Chrivia JC, Kwok RP, Lamb N, Hagiwara M, Montminy MR, et al (1993) Phosphorylated CREB binds specifically to the nuclear protein CBP. Nature 365: 855–859. 8413673 10.1038/365855a0

[86] Wollenick K, Hu J, Kristiansen G, Schraml P, Rehrauer H, et al (2012) Synthetic transactivation screening reveals ETV4 as broad coactivator of hypoxia-inducible factor signaling. Nucleic Acids Res 40: 1928–1943. 22075993 10.1093/nar/gkr978 PMC3300025

[87] Bae MK, Ahn MY, Jeong JW, Bae MH, Lee YM, et al (2002) Jab1 interacts directly with HIF-1alpha and regulates its stability. J Biol Chem 277: 9–12. 11707426 10.1074/jbc.C100442200

[88] Bemis L, Chan DA, Finkielstein CV, Qi L, Sutphin PD, et al (2004) Distinct aerobic and hypoxic mechanisms of HIF-alpha regulation by CSN5. Genes Dev 18: 739–744. 15082527 10.1101/gad.1180104 PMC387414

[89] Claret FX, Hibi M, Dhut S, Toda T, Karin M (1996) A new group of conserved coactivators that increase the specificity of AP-1 transcription factors. Nature 383: 453–457. 8837781 10.1038/383453a0

[90] Jiang Y, Xue ZH, Shen WZ, Du KM, Yan H, et al (2005) Desferrioxamine induces leukemic cell differentiation potentially by hypoxia-inducible factor-1 alpha that augments transcriptional activity of CCAAT/enhancer-binding protein-alpha. Leukemia 19: 1239–1247. 15902299 10.1038/sj.leu.2403734

[91] Yang L, Jiang Y, Wu SF, Zhou MY, Wu YL, et al (2008) CCAAT/enhancer-binding protein alpha antagonizes transcriptional activity of hypoxia-inducible factor 1 alpha with direct protein-protein interaction. Carcinogenesis 29: 291–298. 18024476 10.1093/carcin/bgm262

[92] Zhao XY, Zhao KW, Jiang Y, Zhao M, Chen GQ (2011) Synergistic induction of galectin-1 by CCAAT/enhancer binding protein alpha and hypoxia-inducible factor 1alpha and its role in differentiation of acute myeloid leukemic cells. J Biol Chem 286: 36808–36819. 21880716 10.1074/jbc.M111.247262 PMC3196150

