# Supplementary material for: Cooperativity of Stress-Responsive Transcription Factors in Core Hypoxia-Inducible Factor Binding Regions
Source: PLoS One. 2012 Sep 24;7(9):e45708. doi: 10.1371/journal.pone.0045708 (PMC3454324; doi:10.1371/journal.pone.0045708)
Supplement: Figure S1 — Construction of a background set of control sequences resembling core HIF binding regions. (PDF) [file pone.0045708.s001.pdf]

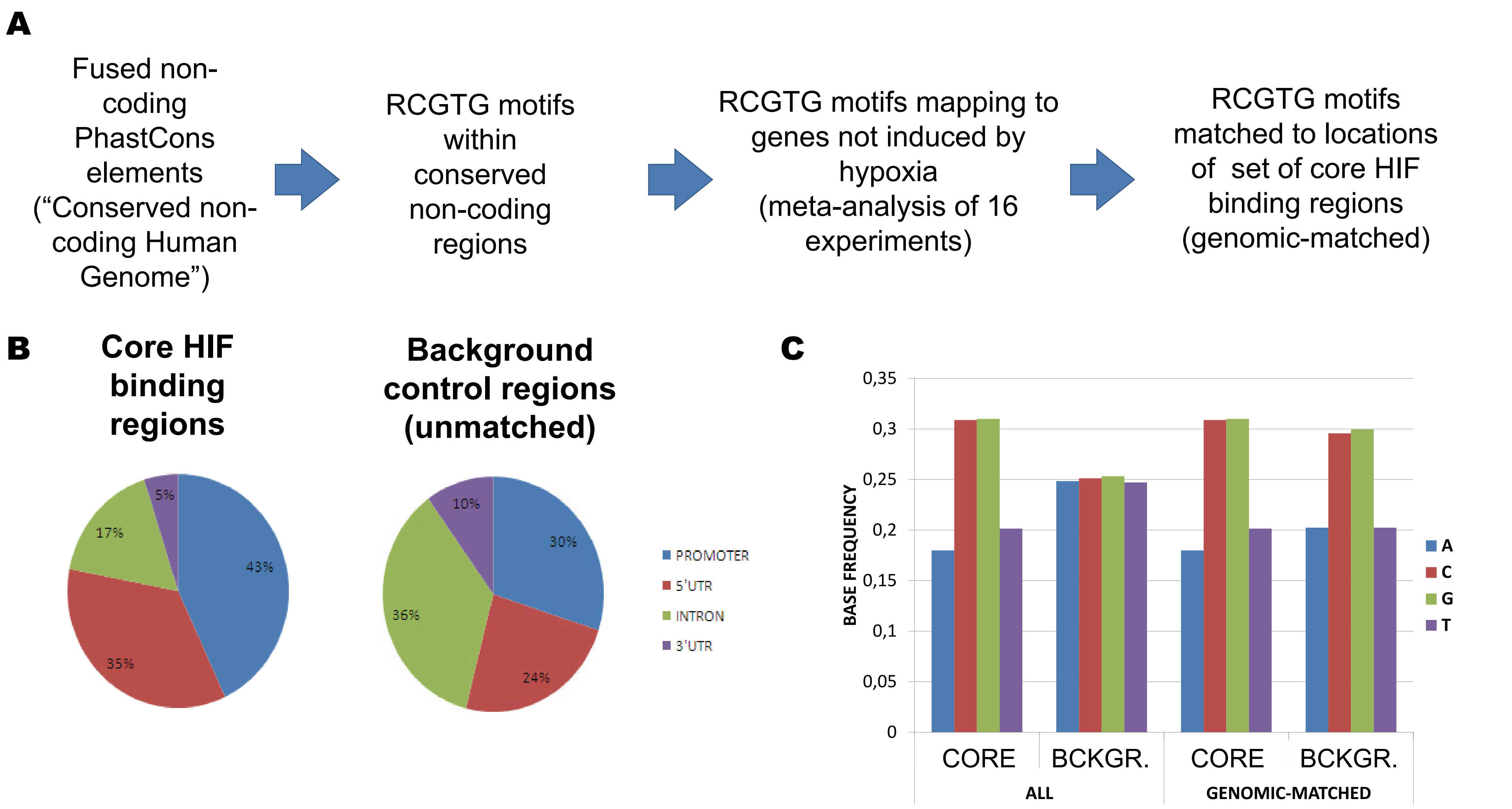

Figure S1: Construction of a background set of control sequences resembling core HIF binding regions. (A) Stepwise schema of the employed strategy **(B)** Pie charts of genomic location distributions relative to the TSS showing significant differences between selected core HIF binding regions and unmatched background controls **(C)** Nucleotide frequencies of core and background (BCKGR.) sets before and after genomic-matching of control sequences.
